# Standardized Solutions of Catecholamines in Intensive Care Medicine: Application, Safety and Economic Aspects

**DOI:** 10.3390/jcm13113070

**Published:** 2024-05-24

**Authors:** Armin Niklas Flinspach, André Mohr, Jahn Wehrle, Kai Zacharowski, Vanessa Neef, Florian Jürgen Raimann

**Affiliations:** 1 Department of Anaesthesiology, Intensive Care Medicine and Pain Therapy, University Hospital Frankfurt, Goethe-University Frankfurt, Theodor-Stern Kai 7, 60590 Frankfurt, Germany; 2 Medical Clinic 2 (Hematology, Oncology, Haemostaseology, Rheumatology, Infectiology), University Hospital Frankfurt, Goethe University, Theodor-Stern-Kai 7, 60590 Frankfurt, Germany; 3 Department of Pharmacy, University Hospital Frankfurt, Goethe University, Theodor-Stern-Kai 7, 60590 Frankfurt, Germany

**Keywords:** critical care, catecholamines, patient safety, safety management, clinical pharmacy service, syringes

## Abstract

**Background/Objectives**: Catecholamines are among those agents that are indispensable in modern intensive care medicine. The rapid availability of hygienically impeccable and correctly concentrated injectable solutions, e.g., for syringe pumps, is becoming more and more important. However, little research has been conducted regarding how the use of catecholamines is distributed in different wards and what options can be used to achieve optimal availability. **Methods**: In a retrospective monocentric study from 2019 to 2022, all continuously applied catecholamines in intensive care units (ICU) and intermediate care units (IMC) were investigated. The focus was on potential optimization by utilizing manufactured ready-to-administer solutions in the context of the economization of patient care. **Results:** Norepinephrine syringes represented 81% of all syringes administered, appearing to be the most frequently used on all wards. Production by the in-house pharmacy showed both financial advantages and an increase in patient safety compared to syringes produced at the bedside. **Discussion:** Increasing numbers of critically ill patients coupled with growing staff shortages and an increased awareness of safety requirements are driving the move towards ready-to-use and ready-to-administer solutions in critical care medicine. In-house manufacturing by hospital pharmacies can be a promising option to optimize processes and improve the economics of patient care. **Conclusions:** Individual calculations of the required catecholamine preparations with regard to possible economic advantages should be carried out in hospitals. In particular, in-house production of ready-to-use and ready-to-administer preparations could significantly increase patient safety and seems to be economically viable.

## 1. Introduction

Catecholamines are indispensable substances in modern critical care medicine. Predominantly epinephrine and norepinephrine, as well as dobutamine, are used in intensive care medicine to maintain or stabilize blood pressure and improve cardiac function [1]. In addition, phosphodiesterase inhibitors, such as milrinone, are increasingly being used for cardiac support to raise inotropic effects. In addition, vasopressin is used as an analogue of antidiuretic hormone and is a distinctly vasoactive substance that increases blood pressure. Today, all modern intensive care units (ICU) usually have a large number of syringe and infusion pumps for the controlled administration of numerous available substances [2].

Catecholamines and the other abovementioned cardiac and circulatory agents stand out due to their high potency and immediate effects but can have deleterious consequences in cases of incorrect dosing. Norepinephrine, epinephrine, vasopressin, dobutamine and milrinone, which are used in intensive care and which we observed, are mainly administered intravenously and mostly continuously due to their short plasma half-lives of a few minutes. However, the determination of dose-adjusted plasma levels in critically ill patients is limited such that an effect-controlled dose adjustment is required [3]. The endogenously produced substances norepinephrine, epinephrine and dobutamine are metabolized in two steps by catechol-O-methyltransferase and subsequently by monoamine oxidase. The metabolism of milrinone and vasopressin to peptides is primarily hepatic. The excretion of the corresponding catecholamine metabolites is predominantly renal [4]. Taking into account these effects, special demands are placed on medical devices with regard to safety requirements, which have been established and increased over the years. Due to the narrow therapeutic index of the aforementioned cardiac and circulatory agents, the highest precautions must be considered for all aspects, including i.a. preparation, hygiene, documentation and application, to avoid incorrect dosing and adverse drug reactions. However, due to progressive economization and a continuing increase in the number of critically ill patients that need to be cared for, existing concepts must also be revamped. The growing challenges to provide medical treatment to patients with severe diseases and considering demographic changes towards more elderly, multimorbid patients will generate a continuously evolving demand for periprocedural intensive care medicine.

While concerted and standardized concentrations of catecholamines (among other agents) are already used, e.g., in the United States and United Kingdom, to avoid errors, this is regrettably lacking in many other countries worldwide [5]. Accordingly, many hospitals still have evolved structures with different concentrations of single agents (including catecholamines) as well as differences in the scaling of infusion flow rates (e.g., mL/min vs. mL/h), even in different wards of the same hospital.

The presence of different in-house catecholamine concentrations and units of administration must be considered as one of the main reasons for dosing errors and associated complications. Furthermore, improperly prepared syringes without standardized procedures can cause microbiological contamination or incorrect dosing ratios, which can lead to serious complications and patient harm [6]. The rate of adverse events in the ICU is reported in various studies to be more than 30%. Corresponding patient harm must be urgently avoided and, if not, may cause considerable follow-up costs in the range of USD 37,000 per case [7].

The pharmaceutical industry has recognized the importance of the safest possible application of drugs and is making an increasing number of prefabricated products available. The majority of commercially available products refer to 50–100 mL ready-to-use (RTU) vials whose contents need to be transferred to suitable containers before application or small-volume, ready-to-administer (RTA) 10 mL emergency syringes with specified concentrations of catecholamine, in particular norepinephrine. RTA syringes designed for continuous infusion, which already contain the desired concentration of catecholamines or other defined agents in compatible carrier solutions, are already dispensed in suitable containers for application and could therefore be used immediately in the ICU without any further steps; these syringes are currently rarely commercially available.

An important benefit of both commercially available as well as hospital- and pharmacy-manufactured RTU and RTA products is the required high quality standards. Quality controls regarding i.a. concentration and microbial purity are extensively performed, and processes used to test the validity of the product’s pharmacology are standardized. Also, the shelf life of the pharmaceutical preparation must be determined based on stability testing. In refrigerated conditions, the shelf life varies from a few weeks to several months (e.g., 18 months); products such as a few licensed RTU solutions, which are commercially available throughout Europe, can be stored at room temperature with European Medicines Agency (EMA) approval.

Economization in health care is leading to several closures of some smaller hospitals in many European countries, especially in urban areas, resulting in an increasing concentration of health care services in fewer hospitals, albeit with a more diversified infrastructure [8,9,10]. The resulting altered infrastructure includes a growing number of major hospitals with in-house pharmacies with the option of manufacturing customized pharmaceutical preparations [11]. The individual preparation of pharmaceuticals has been standard practice in oncology for many years. However, so far, this has been of minor importance in the provision of medication for critically ill patients [12].

For instance, medication errors have already been described in various studies as a cause of adverse events with repetitive deleterious outcomes [13,14,15]. In order to avoid sources of error on wards, the British National Patient Safety Agency (NPSA) called for the use and purchase of RTU and RTA products to improve patient safety as early as 2007. The standardization of preparation instructions and concentrations and the use of RTU and RTA products to reduce medication errors is highly recommended [16,17,18]. The expertise of clinical pharmacists has already proven to be of great advantage in various constellations of therapy for critically ill patients [19]. Although the production of in-house RTU solutions seems just as conceivable and promising as RTA solutions in many respects, RTA solutions offer the following advantages: they are simpler and faster to administer, leading to fewer errors, and require fewer materials (no intermediate packaging). However, this requires the clinical pharmacy to customize the packaging according to internal clinical standards (e.g., syringe pumps and syringes). In particular, it is reasonable to assume that RTA catecholamine products contribute equally to patient safety.

Here, we aim to retrospectively evaluate the use of catecholamines based on real treatment data from different specialized intensive care units, assess the cost–benefit ratio and production costs of the in-house production of 50 mL syringes of manufactured catecholamines and assess the need to evaluate the procurement of corresponding individual pharmacological preparations.

## 2. Materials and Methods

This retrospective observational study was conducted at a tertiary university hospital. The protocol of data acquisition was approved by the ethics committee of Goethe University (#2022-995), and a waiver of written informed consent was approved. This study was conducted in accordance with the Declaration of Helsinki [20]. Due to its retrospective design, this study was not registered in a trial database.

### 2.1. Patient Population and Data Collection

We retrospectively assessed the use of the cardiac and circulatory pharmaceuticals epinephrine, norepinephrine, dobutamine, milrinone and vasopressin during an observational period from 1 January 2019 to 31 December 2022 at the University Hospital Frankfurt.

Catecholamine treatments of all patients >18 years of age, who were treated in one of the university ICUs or intermediate care units (IMC), were included in this study. In order to obtain a data set that is as homogeneous as possible, the data of all operating ICUs participating in the care of critically ill adults were enclosed. Therefore, samples include patients from all internal medicine departments, as well as all neurological and neurosurgical patients and the entire spectrum of modern surgical departments, including cardiac surgery.

All documented changes, initiations and terminations in catecholamines were extracted from the patient data management system (PDMS) Metavision (Version 5.4, iMDsoft, Tel Aviv, Israel). Data were stored in a Microsoft Excel pivot table (Version 365, Microsoft Corp., Redmond, CA, USA) with restricted access. A differentiated evaluation was carried out with regard to each year during the observational period for the abovementioned catecholamines. The data were extracted by the in-house data integration center (DIZ) according to the defined endpoints, number of new syringe pumps documented and dose changes.

### 2.2. Statistical Analysis

No pre-calculation of statistical power was made based on the fact that the sample size was based on the maximum number of records available from the survey period. The categorical variables are presented as counts and percentages. All statistical tests were two-tailed, and results with *p* < 0.05 were considered statistically significant. All analyses were performed with SPSS (IBM Corp., Version 29, Chicago, IL, USA).

## 3. Results

Over the study period, 96,623 syringes were applied via syringe pumps in the university ICUs and IMCs. There were differences between the applied catecholamines due to differences in the spectrum of patients that were cared for in the respective specialized ICUs (Table 1).

**Figure 1:**
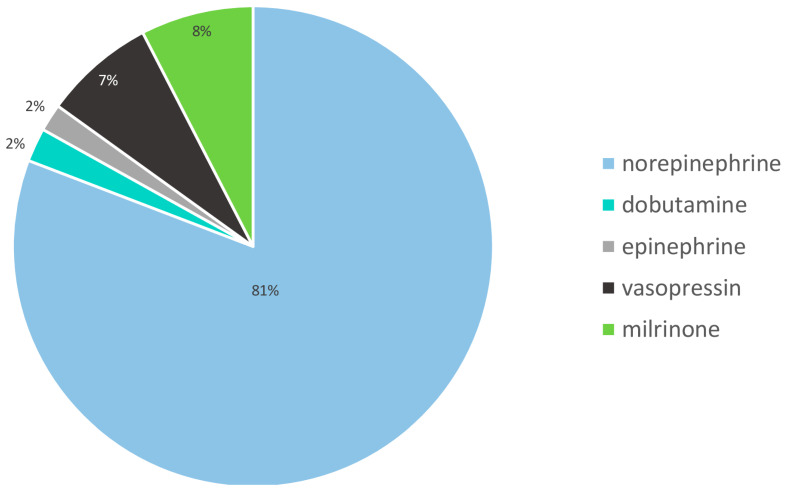
Distributed catecholamine 50 mL syringes. The administered catecholamine 50 mL syringes contained the following concentrations: norepinephrine, 100 µg/mL (5 mg/50 mL); dobutamine, 5 mg/mL (250 mg/50 mL); adrenaline, 100 µg/mL (5 mg/50 mL); vasopressin, 0.5 IU/mL (20 IU/40 mL); milrinone, 0.2 mg/mL (10 mg/50 mL). Abbreviations: mg, milligram; mL, milliliters; µg, microgram; IU, international units.

We found that norepinephrine was the most frequently used catecholamine among those administered (Figure 1).

During the observed period, the in-house hospital pharmacy dispensed a median of 660 syringes per week with 50 mL of norepinephrine at a dosage of 100 µg/mL. This corresponds to a quantity of 137,280 syringes. Of the prepared syringes, 104,463 syringes were delivered to the intensive care units, and 69.9% of these were used to supply patients in the adult ICU. In addition, the syringes had already been supplied to the operating theatre.

Our in-house economic analysis of the production costs, consisting of material, raw substance, storage, delivery and warehousing costs, showed that production, due to the provision of various existing structures (established analytics, microbiological quality assurance, pharmaceuticals distribution, etc.), achieved a cost advantage over commercially available products. Our analysis showed advantages in terms of staff working time as well as consumption of materials, both in terms of pure preparation on the ward and preparation of preformed, ready-to-use solutions. The in-house pharmacy preparation included the preparation of a norepinephrine stock solution in a reusable container with a volume of up to several liters, as required. The subsequent transfer to 50 mL syringes was carried out using a disposable filling system under sterile conditions. Subsequent labelling and suitable packaging completed the process of delivery to the ward.

**Figure 2:**
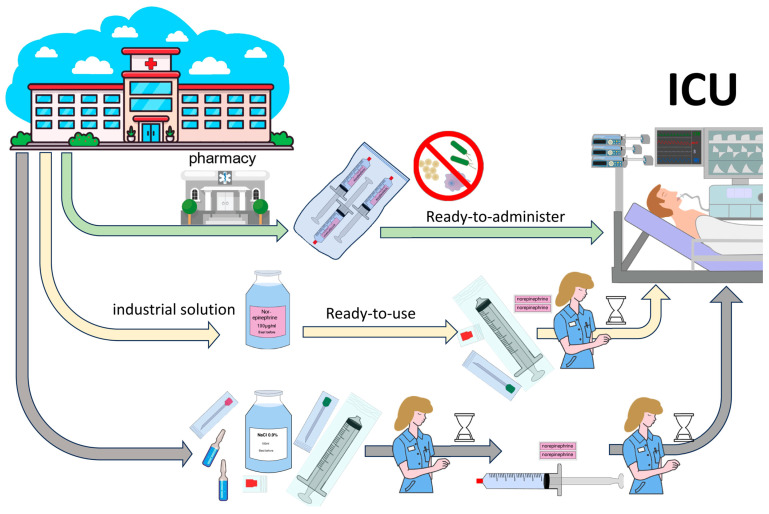
Graphical illustration of the labor-related steps regarding the different options of catecholamine preparation. Illustration of the different possibilities of hospital workflow to provide catecholamines, from the ready-to-administer solution produced in sterile conditions from an in-hospital pharmacy to prepared industrial catecholamine solutions already correctly concentrated as ready-to-use, as well as the complete bedside preparation of a correctly concentrated catecholamine solution and preparation of a dispensable syringe. Abbreviations: ICU, intensive care unit.

Application of an RTU solution requires bedside aspiration from the vial into a 50 mL syringe by a qualified nurse using an aspiration cannula, followed by appropriate labelling at an aseptic workstation. Individual aseptic preparation using a norepinephrine concentrate requires precise removal of the concentrate from the vial using an aspiration cannula, followed by a precise adjustment of the desired concentration through the addition of a diluent solution and appropriate mixing. This stock solution requires aseptic dispensing into a 50 mL syringe and final labelling (illustrated in Figure 2). By implementing RTA/RTU products, 2.5 min per preparation can be saved. These potential savings from reduced work personnel costs were not included in the economic analysis. On average, despite fluctuating unused syringes (discard) and fluctuating purchase costs, a substantial cost benefit was achieved, which is equivalent to an in-house production price of consistently less than 50% of the cost calculated by RTU or commercial RTA. During the study period between 2019 and 2023, various global events occurred, mainly the COVID-19 pandemic, the trade conflicts between the United States and China, and the war in Ukraine, which led to significant fluctuations in the availability of the active pharmaceutical ingredients and their purchase prices. The cost fluctuations occurred several times within a few weeks, often with a multiplication of the prices. However, as the purchase prices of syringes and diluent solutions in particular are relatively inexpensive and apply equally to all forms of administration (bedside preparation, RTU and RTA), the economic price advantages were always apparent.

## 4. Discussion

Treatment with catecholamines, especially norepinephrine, is an inherent part of modern intensive care. [21,22] With regard to the pharmaceutical preparation quality, especially for highly critical drugs such as catecholamines, as few compromises as possible are acceptable. However, especially in high-stress situations, fluctuations in the concentration of bedside preparations are to be expected, and microbiological contamination is more probable. These exemplary data from a university hospital of maximum care show a high consumption of appropriately equipped catecholamine 50 mL syringes. In particular, the quantity of the frequently used catecholamine norepinephrine stands out. This fact can be explained by recommendations for norepinephrine as a first-choice treatment, especially in sepsis [23]. Other substances, such as dobutamine, have been increasingly abandoned, while new therapeutic pharmacological approaches, such as therapies with vasopressin, are still infrequently applied [24,25]. Correspondingly, in our cohort, norepinephrine was applied across all intensive care subdisciplines with an extremely high frequency of use (more than 80%) among related catecholamine prescriptions.

Syringes that have already been prefilled (RTA) have a relevant time benefit in terms of availability in bedside care and, in particular, in terms of safety regarding administering the correct dose. This also provides time savings in terms of nursing time, which can benefit the primary care of the critically ill patient [26]. This makes it worthwhile to prepare such syringes for use in maximum care hospitals. RTA injection solutions or syringes of the most commonly used catecholamines also feature a more precisely traceable dosage and a hygienically proven preparation under sterile conditions following preparation in a pharmacy environment, where compliance with international standards is high compared to individual bedside preparation [27,28,29,30]. This is a great advantage for rapid and safe patient care. Apart from all safety-relevant aspects, it must be considered that the processes of supply and manufacturing in the hospital pharmacies result in enormous time savings for the intensive care staff. Similar benefits could be achieved with the direct purchase of manufactured RTA catecholamine syringes or ready-to-use infusion solutions with specified concentrations. Due to the realization of the aforementioned advantages, the number of RTU and RTA preparations made available by national pharmaceutical distributors has recently risen, but this increase has been limited [31]. In this respect, it is recommended to perform a cost analysis for each hospital with regard to the existing consumption and the available resources, taking into account individual circumstances, such as capacity of the hospital pharmacy, local facilities, number of ICUs and delivery intervals. In addition to the number of available personnel resources, employee satisfaction regarding their assigned tasks is also relevant.

Therefore, focusing solely on the resources of the ICU in charge is too shortsighted. Hence, the economically advantageous relocation of production to a pharmacy must also take this human resource into account. The fact that the assembly line production of large batches of ready-to-fill syringes is a monotonous activity and the need for varied and demanding activities are contradictory ideas at this point. This is a relevant aspect in the context of employee motivation and should not be ignored. Approaches to partial or complete robot-assisted assembly have already been implemented elsewhere but have proven to be far less economical in terms of the high unit numbers required compared to the personalized preparation of oncology drugs [32]. It remains to be seen to what extent the rapid advances in robotics will change this situation in the near future. However, it can be anticipated that further optimizations will also lead to corresponding innovations that will become more attractive even in smaller units, such as local hospital pharmacies.

However, it should be kept in mind that the sole desire for a supply of medicines through in-house pharmacies, particularly to the extent outlined with RTA pharmaceuticals, is not always feasible. Due to the high quality requirements, the production entails a series of structural, technical and personnel-related conditions that must be fulfilled. In addition to a specially designed sterile room or laminar air flow workstation, the pharmacy also needs appropriate filling equipment to ensure that the required number of syringe pumps can be filled.

The importance of having an accurately prepared syringe of life-saving drugs such as norepinephrine quickly and reliably available, especially in critical situations, is increasingly considered to be of high safety relevance [33]. Errors in terms of hygiene or concentration deviations increase under stressful work situations. A study carried out in the U.S. showed that the care of 20 patients in the ICU required 1.11 h of daily working time for the individual preparation of syringe pumps [34].

In our data, there are no applications of the vasoactive substances dopamine, angiotensin II and phenylephrine. The use of these substances has been discussed for many years and is still researched somewhat but not applied in our clinic and has been abandoned by the majority of experts [35].

A limitation of this study is that it is a monocentric investigation, and thus, the data we collected can only accurately reflect the situation at the center the data were collected from. Moreover, the data were analyzed retrospectively, which entails corresponding design weaknesses and allows extremely limited statements regarding price differences at individual time points. Moreover, the public disclosure of detailed unit pricing as well as disclosing associated quantity discounts is subject to restrictions, some of which are contractually fixed. Specifically, producers and retailers often offer extensive discounts based on distinct quantity scales and delivery guarantees, but individual discounts also exist and are commonly kept secret [36]. However, current critical global events, including global trade restrictions, can quickly lead to extreme changes in the market situation with regard to the availability and price of required pharmaceuticals and sterile goods, which were not reflected in our work.

## 5. Conclusions

Catecholamines are extensively used in the care of critically ill patients in all adult ICUs and IMCs. The catecholamine norepinephrine stands out in terms of volume due to its prominent role in blood pressure stabilization. Preformulated RTU or RTA products, especially commonly used emergency drugs such as norepinephrine, are increasingly required for safety reasons. The in-house production and dispensing of corresponding drugs by hospital pharmacies can reduce the workload of intensive care staff and may be associated with economic benefits, so a hospital-specific assessment should be considered on an individual basis.

## Figures and Tables

**Table 1:** Number of patients treated and catecholamine syringes applied.

	Anaesthesiological–Surgical ICU	Internal Medicine ICU	Neurology–Neurosurgical ICU	Overall
Patients [*n*]	10,912 (40.3%)	9833 (36.3%)	6347 (23.4%)	27,092
Norepinephrine [50 mL; 100 µg/mL]	39,093 (50.1%)	22,436 (28.7%)	16,562 (21.2%)	78,091
Epinephrine [50 mL; 100 µg/mL]	1303 (73.2%)	390 (21.9%)	86 (4.8%)	1179
Vasopressin [40 mL; 0.5 IU/mL]	4263 (58.9%)	2176 (30.1%)	795 (11.0%)	7234
Dobutamine [50 mL; 5 mg/mL]	297 (13.6%)	1878 (85.8%)	13 (0.6%)	2188
Milrinon [50 mL; 0.2 mg/mL]	6354 (86.7%)	830 (11.3%)	147 (2.0%)	7331
Overall	51,310	27,710	17,630	96,623

Tabular overview of the administered count of catecholamine syringes during the study period, as well as the corresponding number of patients treated, with a breakdown according to primary specialized intensive care unit. Abbreviations: ICU, intensive care unit; mg, milligram; mL, milliliters; µg, microgram; IU, international units.

## Data Availability

The data cannot be shared publicly. The datasets generated and/or analyzed during the current study are not publicly available due to national data protection laws but are available upon reasonable request from the corresponding author or via the data protection officer of the University Hospital of Frankfurt.
